# Rapid detection of bovine adipose tissue using lateral flow strip assay

**DOI:** 10.1002/fsn3.322

**Published:** 2015-12-12

**Authors:** Yun‐Hwa P. Hsieh, Kamil Gajewski

**Affiliations:** ^1^ Department of NutritionFood and Exercise SciencesFlorida State UniversityTallahasseeFlorida32306; ^2^ Flat 12 Hampton Court, Batavia RoadLondonSE14 6AQUnited Kingdom

**Keywords:** Adulteration, immunoassay, lipids, proteins

## Abstract

Currently no rapid immunoassays are developed to identify the species content of fat tissue in mixtures. We report a simple protocol enabling the effective detection of bovine fat in highly processed materials using a lateral flow (LF) immunoassay which targets a ruminant‐specific muscle protein. A portion (50 gm) of muscle‐free fat samples was rendered to separate the molten fat from the proteinaceous residue, then soluble proteins were extracted from the solid residue with 0.5 mol/L NaCl for the LF analysis. The assay could detect 2% bovine fat‐in‐pork fat, 1% bovine fat‐in‐porcine meat‐and‐bone meal, and 0.5% bovine fat‐in‐soy meal mixtures. Rendered bovine fat could be detected up to 213°C. These results demonstrate that low levels of bovine fat tissue can be detected in processed materials using an immunoassay based on the presence of the muscle protein which serves as a species marker in the fat tissue.

## Introduction

Animal fat is an important by‐product for the meat industry. Fat tissue is often used in the manufacture of processed meat products such as sausages, frankfurters, and canned meats, and in nonmeat food products to improve the flavor or texture. Animal fat is also used in rendering plants to produce a fat commodity (such as yellow or white grease, tallow, or lard) and in protein meals such as meat‐and‐bone meal (MBM) for animal nutrition.

The undeclared use of bovine fat in processed samples is a concern for a number of reasons. For example, food containing undeclared ingredients derived from bovine sources may be a serious problem for adherents to religions such as Hinduism, and for vegetarians. There are also people who refrain from consuming fats from ruminants (cattle, sheep, and deer) for health reasons because their unhealthy fatty acid profile has been implicated in chronic diseases. The adulteration of lard with tallow has been reported because of the relatively lower price of tallow (Vaclavik et al. 2011) and tallow may also present a health risk due to the possibility that it may carry the infectious agent – prion – that causes the spread of transmissible spongiform encephalopathies (TSEs) such as bovine spongiform encephalopathy (BSE), or so‐called “mad cow disease” (ECSSC 1999). The TSE risk from fat is mainly due to protein residues in the end‐product. To ensure the safe use of ruminant fat in animal nutrition in Europe and the United States, the regulations require that the maximum concentration of residual insoluble proteinaceous impurities does not exceed 0.15% (Commission Regulation 1774/2002 2002; 21 C.F.R. § 589, 2008).

As intentional and accidental food/feed adulteration and contamination has become a serious problem worldwide (Hsieh 2006), in order to protect consumers from these risks associated with food fraud and the ambiguous labeling of fat ingredients in both food and feed, a number of analytical methods have been proposed in the literature for identifying the origin of the animal fat. These methods include (1) fat‐based methods such as chromatography (Marikkar et al. 2005; Szabó et al. 2007), spectroscopy (Beattie et al. 2007; Martín et al. 2007; Abbas et al. 2009; Motoyama et al. 2010; Che Man et al. 2011), and differential scanning calorimetry (Aktas¸ and Kaya 2001; Marikkar et al. 2002), and (2) deoxyribonucleic acid (DNA)‐based methods (Montiel‐Sosa et al. 2000; Aida et al. 2007, 2011; Martín et al. 2007). However, all these methods involve the use of expensive instruments operated by highly skilled technicians and requiring complicated data analyses, and focus almost exclusively on the speciation of raw fat present in copious amounts. They are no rapid and effective methods developed for analyzing low levels of target fat tissue in processed sample mixtures.

As yet, there are no reports in the literature of the development of protein‐based rapid immunoassays specifically targeted for fat analysis. However, one study (Bellorini et al. 2005) evaluated the potential of FTIR (Fourier Transform Infrared spectroscopy), GC, PCR techniques, and a commercial single‐step lateral flow (LF) immunochromatographic assay (Neogen's *AgriScreen for Ruminant in Feed*, later renamed *Reveal for Ruminant in Feed*) for the identification of ruminant fat and its differentiation from nonruminant fats. Of the four methods tested, only immunoassay and PCR were able to identify the species of the fat. They were also the only techniques capable of identifying low concentrations of tallow in a mixture of fats typical of those prepared by the rendering industry. Although FTIR and GC‐MS could differentiate between pure fat samples, they exhibited only a limited ability to identify the animal species or even the animal class from which the fat(s) originated (Bellorini et al. 2005).

Although the immunoassay used in the study by Bellorini et al. (2005) was performed with the rapid LF assay, the sample preparation, which involved three cycles of centrifugation and organic solvent extraction prior to the LF analysis, were both laborious and time consuming. The main goal of this study was therefore to evaluate the effectiveness of an improved simple protocol of sample preparation to rapidly separate the proteinaceous residue from melted fat by rendering to facilitate the detection of bovine fat tissue in a fat mixture prior to the LF analysis. The same ruminant‐specific LF assay used by Bellorini et al. (2005) was used for comparison. This simplified protocol was utilized to accomplish three specific objectives of this study: (a) to compare the performance of two related commercial immunoassay test kits: Neogen's *Reveal for Ruminant in Feed* and *Reveal for Ruminant in MBM* for bovine fat detection; (b) to investigate the effect of temperature on the assay signal; and (c) to determine the assay detection limits in both fat mixtures and feedstuffs.

## Materials and Methods

### Sample preparation

Bovine and porcine adipose tissues trimmed from intact pieces of meat were purchased from a local supermarket. After removing any visible muscle and connective tissues, the fat tissue (bovine or porcine) was minced into paste‐like small pieces, mixed well, and a portion (50 gm) rendered in a cooking pan on low heat for 7 min. To study the effect of heating temperature, four equal portions of fresh beef fat tissue were rendered separately for 7, 9, 12, and 15 min, respectively. The temperature of the melted fat was monitored for each sample using a metal Traceable^®^ thermometer (Fisher Scientific, Fair Lawn, NJ). After separating the fat from the solid residue, which contains proteins, the hot molten fat portion was poured off and the insoluble residue absorbed on a paper towel to remove the adhered liquid fat while each sample was still warm. The solid residues were then soaked in petroleum ether (250 mL) for an hour to dissolve residual fat, and then drained, air‐dried and stored in glass vials at 4°C until use.

Four kinds of samples spiked with beef fat were prepared for the fat analysis as follows: (1) fresh pork fat was spiked with beef fat at 1%, 2%, 5%, and 10% (w/w) levels in separate sterile sample bags, sealed and mixed thoroughly by hand for a minute to ensure homogeneity. Pure (0%) beef fat and pure (100%) pork fat were prepared separately as the positive and negative controls. The mixed raw samples were then rendered as described above; (2) rendered beef fat residue was spiked in pork MBM (donated from a commercial source) at levels of 0%, 1%, 2%, 5% and 10% (w/w); (3) beef fat residue was spiked in soybean meal (Soy Best/Grain States Soya, Inc., West Point, NE) at levels of 0%, 0.5%, 1%, 2%, and 5% (w/w); and (4) rendered beef fat residue was spiked in rendered pork fat residue at levels of 0%, 0.5%, 1%, 2%, 5%, and 10% (w/w).

### Sample analysis

The soluble protein extraction of the dry residue samples and the LF assay procedures were conducted according to the manufacturer's instructions for the *Reveal for Ruminant in MBM* and the *Reveal for Ruminant in Feed* LF assay kits (Neogen Co., Lansing, MI). Each dry sample was mixed 1:10 (w/v) with the extraction solvent provided in the kit and the tubes containing the sample mixture heated in boiling water for 10 min, after which approximately 0.5 mL of each sample extract was transferred to a 1.5 mL‐microcentrifuge tube. A test strip was then placed into each sample tube and allowed to stand at room temperature for 10 min. The sample extract is wicked through the reagent zone of the strip, which contains antibodies specific for heat stable ruminant muscle protein conjugated to colored particles. If the sample contains protein from ruminant by‐products, the ruminant protein is captured by the conjugated antibodies and the resulting ruminant protein‐antibody‐particle complex is then wicked to a sample zone containing a second antibody specific for the ruminant muscle protein. This zone captures the complex, allowing the particles to concentrate and form a visible line (the “sample line”) if ruminant by‐product is present. The strip also contains a control zone where an immune complex present in the reagent zone is captured by a third antibody to form a visible line (the “control line”), which always forms regardless of the presence of the target antigen to signal that the assay is valid by confirming that the capillary action of the strip is adequate. The result is recorded once a clear control line is observed on the test strip, which normally takes about 10 minutes. The color intensity of the Test Line developed is proportional to the target ruminant protein content in the sample. For this study, all experiments were performed in triplicate and all experiments were repeated.

## Results

### Detection of rendered bovine fat in porcine MBM

In order to compare the performance of the commercial *Reveal for Ruminant in Feed* assay with its sister product *Reveal for Ruminant in MBM* for bovine fat detection, both LF assay kits were used to analyze rendered samples of porcine MBM samples spiked with bovine fat at five levels (0%, 1%, 2%, 5%, and 10%, w/w). Their assay signals are summarized in Table 1. Both assay kits detected rendered bovine fat in pork MBM with no cross reaction with pure pork MBM. The *Reveal for Ruminant in Feed* assay appeared to be more sensitive as it detected down to 1% (w/w) of rendered bovine fat in pork MBM, whereas the *Reveal for Ruminant in MBM* assay was only able to detect 2% or higher levels of bovine fat in porcine MBM. From the product specification information provided by the manufacturer, the sensitivity of the former is 1% in feed and feed supplements, and of the latter 2% in MBM, so our results support the claimed detection limits. Interestingly, although the *Reveal for Ruminant in MBM* assay is specifically designed for the qualitative analysis of ruminant by‐products in MBM, the *Reveal for Ruminant in Feed* assay actually achieved a more sensitive detection level in MBM in our study. Based on the higher sensitivity of the assay, the *Reveal for Ruminant in Feed* assay was thus selected for the subsequent experiments conducted for this study described below.

**Table 1:** Detection of bovine fat in porcine MBM using two ruminant‐specific lateral flow assay kits

Assay kit	Percentage rendered spiked beef fat in pork MBM (w/w)				
	0%	1%	2%	5%	10%
Reveal for Ruminant in Feed	−	+	++	++	+++
Reveal for Ruminant in MBM	−	−	+	++	+++

−, negative result; +, positive result; ++, strongly positive result; +++, very strongly positive result.

### Detection limits of the assay in fat mixtures and in a feedstuff

Because soybean meal is commonly used as the major plant protein source in animal feed, we determined the detection limit of the LF assay for rendered tallow spiked soybean meal at levels of 0%, 0.5%, 1%, 2%, and 5% (w/w). As shown in Table 2, the rendered bovine fat solids could be detected in the amount as low as 0.5% (w/w) and exhibited no cross reactions with pure soybean meal (0% sample).

**Table 2:** Detection of spiked bovine fat in soybean meal or in porcine fat (*n* = 3) using *Reveal for Ruminant in Feed* lateral flow strip assay

Spiked sample (w/w)	Spiking levels					
	0%	0.5%	1%	2%	5%	10%
Rendered beef fat in soybean meal	−	**+**	++	+++	+++	NT
Rendered beef fat in rendered pork fat	−	−	**+**	++	++	+++
Fresh beef fat in fresh pork fat	−	NT	−	**+**	++	+++

−, negative result; +, positive result; ++, strongly positive result; +++, very strongly positive result; NT, not tested.

Among animal fats, pork, and beef fats are used most commonly. To determine the detection limit of the LF assay for bovine fat in porcine fat, samples were prepared in two ways: (a) spiking fresh porcine fat tissues with fresh bovine fat, and then rendered the admixture for subsequent protein extraction; and (b) mixing rendered bovine fat solid residue with rendered bovine fat residue, followed by protein extraction. Both sets of samples were prepared at 6 bovine fat spiking levels of 0%, 0.5%, 1%, 2%, 5%, and 10% (w/w). A detection limit of 2% of bovine fat in porcine fat was obtained in fresh tissue mixtures, but this dropped to 1% in rendered tissue mixtures (Table 2). These results indicate that using a simplified fat‐protein separation method allows at least 2% bovine fat to be detected in various forms of mixtures with porcine fat, which is better than the detection limit reported by Bellorini et al. (2005) for the same assay, who found that 5% of tallow could be detected in a mixture with other fats.

### Effect of rendering temperature on the reaction signal of the assay

**Figure 1:**
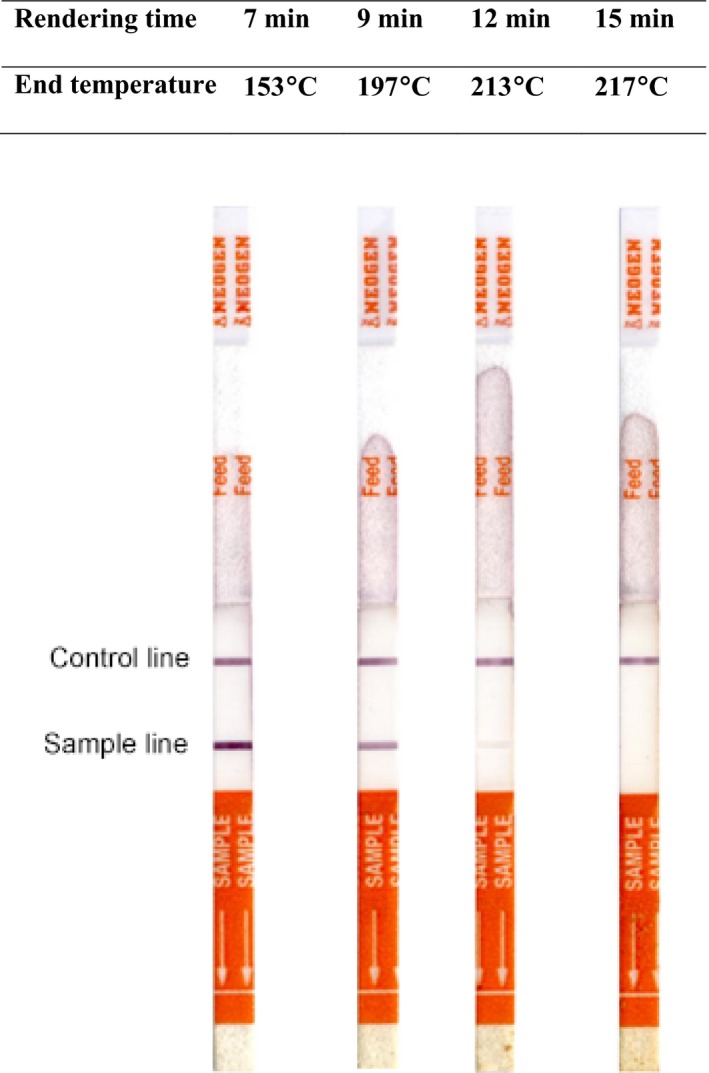
The effect of rendering temperature on the assay reaction signal. Extracts of soluble proteins were obtained from rendering muscle‐free bovine adipose tissue and tested with lateral flow strips. The control line confirms the validity of the assay; the sample line reveals the presence of the target bovine muscle protein in the sample.

The effect of the fat rendering temperature on the assay signal was investigated. Here, small portions (50 gm) of bovine adipose tissue were rendered for 7, 9, 12, and 15 min and the temperature of the rendered fat sample recorded at the end of each rendering time, namely 153.4°C, 197°C, 213°C, and 217°C, respectively. The LF assay results for the rendered bovine fat samples are summarized in Figure 1. A positive sample line indicating the presence of the antigenic protein was observed for bovine adipose tissue samples rendered for 7, 9, and 12 min, but not for the 15 min sample. The overall color of the band formed decreased with increasing rendering time, and hence temperature. Smoke and a harsh smell appeared for the sample rendered to 217°C, at which temperature the fat reaches its smoking point. Only a faint band was visible for the sample rendered for 12 min (213°C) and there was no visible band for the sample rendered for 15 min (217°C), indicating the limit of the thermal stability of the epitope on the target antigenic protein, which exhibited a positive reaction up to 213°C.

## Discussion

Current methods for fat species identification or fat species content determination mainly utilize either fat‐based or DNA‐based approaches. Fat‐based methods rely on subtle differences in the chemical or physical nature of different animal fats to identify their species origin, whereas DNA‐based methods detect species‐dependent differences at the genetic level; both approaches involve laborious fat or DNA extraction and sophisticated instrumental analysis. No protein‐based immunoassays are available that provide an economic and rapid analysis for this purpose. As the previous report on the successful use of the *Reveal for Ruminant in Feed* LF assay for tallow detection in feed (Bellorini et al. 2005) revealed, protein‐based immunoassays offer a feasible approach to analyzing the target proteins present in adipose tissue as these exhibit species‐dependent differences at the protein level. These researchers demonstrated that the target ruminant‐specific muscle antigen is present in adipose tissue in sufficient amounts to achieve a relatively low detection limit of 5% tallow in a mixture of other fats in rendered feed materials (Bellorini et al. 2005).

Adipose tissue is a kind of loose connective tissue composed of mature adipocytes, fibroblasts, immune cells, adipose tissue matrix, and blood vessels. Approximately 60–85% of the weight of adipose tissue is lipid, with 90–99% being triglyceride. The remaining weight of adipose tissue is composed of water (5–30%) and proteins, mainly collagen (2–3%) (Albright and Stern 1998). Adipose tissue has been shown to secrete contractile muscle proteins such as myosin, tropomyosin‐2, tropomyosin *α*‐3, and tropomyosin *α*‐4, all of which have been detected in human and porcine adipose tissues (Ahmed et al. 2010; Rosenow et al. 2010). Adipose tissue also secretes different types of proteins that play important roles in homeostasis and metabolism. A number of proteins, such as cytokines and cytokine‐related proteins, chemokines, other immune‐related proteins, proteins involved in the fibrinolytic system, and enzymes involved in steroid metabolism have all been reported as being secreted in adipose tissue (Kershaw and Flier 2004; Rosenow et al. 2010).

The sample preparation for immunoassays entails the extraction of water‐soluble protein material from the adipose tissue. Methods for protein separation and extraction from adipose tissue are abundant in the literature for research into obesity and obesity‐related diseases (De Taeye et al. 2010; Sajic et al. 2011), fat deposition in livestock (Gondret et al. 2012), and to enforce BSE labeling laws (Zasadny and Kwiatek 2006). Although these methods are useful for their designated purposes, they all suffer from limitations that make them unsuitable for use as a protein extraction protocol for immunoassays. These limitations include the use of organic solvents and chemicals (denaturants, chelators, reducing agents) that may affect the epitopes (antigen–antibody binding sites), and/or the use of detergents that may affect protein recovery or be incompatible with subsequent protein analytical techniques. In addition, most of these extraction methods tend to be laborious and time consuming, or involve the use of specialized clean‐up kits that add to the cost. In the study by Bellorini et al. (2005), in order to separate the solid protein impurities present in tallow from the melted fat portion, the authors applied three cycles of centrifugation to the previously molten fat together with the addition of organic solvent (hexane).

In this study, we used an improved method for the separation of solid protein residues from molten fat that avoids the need to use multiple cycles of centrifugation and hazardous chemicals. All fat samples were prepared using a simple rendering procedure. Given the thermal stability of the antigenic protein present in adipose tissue, the samples can be heated to separate the melted fat from the solid proteinaceous residue, after which the water‐soluble proteins can be extracted using an aqueous buffer for the subsequent LF analysis. By using this simplified method, the detection sensitivity of the immunoassay achieved the manufacturer's stated low detection limit of 1–2% muscle‐free adipose tissue in feed, MBM, and meat samples. There are no other reports in the literature of methods capable of sensitively identifying the species content of processed fat samples at such low levels in a mixture. It should be noted that neither of the Neogen LF assays tested is intended for bovine fat detection but instead targets muscle tissue in feed and MBM samples using a pair of ruminant‐specific antibodies as the antigen probe. Although muscle has a much higher protein content than adipose tissue, our results provide strong evidence that our simplified protocol not only can extract higher amount of the species marker proteins from fat tissue which improves the assay sensitivity (1–2% vs. 5%), but also shorten the sample preparation time for a rapid immunoassay.

Bellorini et al. (2005) reported that the ruminant LF assay was capable of detecting the presence of ruminant proteins in MBM materials that had been heat treated up to 138°C. Our examination of the effect of rendering temperature on the reaction signal of the LF assay revealed that the assay was effective even when the rendered fat samples reached temperatures as high as 213°C. The discrepancy between their result and ours in the upper temperature limit is mainly due to the difference in sample used. A temperature of 138°C was reached under pressurized treatment in the MBM sample, a mixture of muscle, fat, and bone, whereas the pure fat material tested in our study reached a temperature of 213°C. The specific heat of fat (0.45 cal/gm°C) is less than half of that of water (1 cal/gm°C), thus causes fat to reach a higher temperature than an equal amount of meat which contains a relatively high percentage of water, under the same heating conditions. Nevertheless, this rendering fat sample preparation method reveals the actual upper limit temperature at which the antigen in the tissue can be detected. This result also indicates that although the assay's target protein is heat stable up to the smoking point of tallow (420°F/216°C), this would not affect the qualitative analysis of the target fat under normal heating conditions, however, severe or prolonged heat treatment would decrease the intensity of the signal, and thus the sensitivity of the assay.

In conclusion, as a simplified sample preparation protocol to separate molten fat from its proteinaceous residue, the rendering process employed in this study not only shortened the sample preparation time but also enhanced the target protein extraction from fat tissue as demonstrated by increased assay sensitivity which lowers the detection limit. This technique avoids the use of centrifugation and organic chemicals that may interfere with immunoassays. Our results also shed a light that the target ruminant species marker muscle protein also present in sufficient amount in ruminant adipose tissue. Although both commercial LF assays used in this study have been designed and marketed for the detection of ruminant muscle content in feed materials, they can also be used to detect the ruminant content of raw and processed adipose tissue in a food and feed materials. The immunoreactivity of the target antigenic protein, however, can be affected by excessive heat treatment conditions. This is the first report that species origin of a fat sample can be identified at such low levels in a highly processed mixture based on the recognition of these species marker proteins. This study has shed a light that a rapid immunoassay is feasible for a cost‐ and labor‐effective and sensitive determination of fat species content in highly processed samples. The development of suitable immunoassays for fat speciation and quantification based on the recognition of stable species‐specific antigen(s) is now underway in our laboratory. It should be noted that as immunoassays are based on the detection of protein through the strong affinity binding of the target antigenic protein with a specific antibody, an immunoassay would not be capable of identifying a highly refined fat sample with extremely low residual protein content.

## Conflict of Interest

The authors declare that the research was conducted in the absence of any commercial or financial relationships that could be construed as a potential conflict of interest.

## References

[fsn3322-bib-0001] Abbas, O. , J. A. Fernández Pierna , R. Codony , C. von Holst , and V. Baeten . 2009 Assessment of the discrimination of animal fat by FT‐Raman spectroscopy. J. Mol. Struct. 924:294–300.

[fsn3322-bib-0002] Ahmed, M. , M. J. Neville , M. J. Edelmann , B. M. Kessler , and F. Karpe . 2010 Proteomic analysis of human adipose tissue after rosiglitazone treatment shows coordinated changes to promote glucose uptake. Obesity 18:27–34. 19556978 10.1038/oby.2009.208

[fsn3322-bib-0003] Aida, A. A. , Y. B. Che Man , A. R. Raha , and R. Son . 2007 Detection of pig derivatives in food products for halal authentication by polymerase chain reaction–restriction fragment length polymorphism. J. Sci. Food Agric. 87:569–572.

[fsn3322-bib-0004] Aida, A. A. , Y. B. Che Man , C. M. V. L. Wong , A. R. Raha , and R. Son . 2011 Specific polymerase chain reaction (PCR) analysis of raw meats and fats of pigs for Halal authentication. Middle‐East J. Sci. Res. 7:1008–1013. 10.1016/j.meatsci.2004.06.020 22062638

[fsn3322-bib-0005] Aktaş, N. , and M. Kaya . 2001 Detection of beef body fat and margarine in butterfat by differential scanning calorimetry. J. Therm. Anal. Calorimetry 66:795–801.

[fsn3322-bib-0006] Albright, A. L. , and J. S. Stern . 1998 Adipose tissue *in* Fahey T. D., ed.Encyclopedia of Sports Medicine and Science, Internet Society for Sport Science Available at http://www.sportsci.org/encyc/adipose/adipose.html (accessed 14 July, 2015).

[fsn3322-bib-0007] Beattie, J. R. , S. E. J. Bell , C. Borggaard , A. M. Fearon , and B. W. Moss . 2007 Classification of adipose tissue species using Raman spectroscopy. Lipids 42:679–685. 17486383 10.1007/s11745-007-3059-z

[fsn3322-bib-0008] Bellorini, S. , S. Strathmann , V. Baeten , O. Fumière , G. Berben , S. Tirendi , et al. 2005 Discriminating animal fats and their origins: assessing the potentials of Fourier transform infrared spectroscopy, gas chromatography, immunoassay and polymerase chain reaction techniques. Anal. Bioanal. Chem. 382:1073–1083. 15933852 10.1007/s00216-005-3213-5

[fsn3322-bib-0009] 21 C.F.R. § 589 . 2008 Code of Federal Regulations Title 21 Part 589. Substances prohibited from use in animal food or feed. Fed. Reg. 73, 589.2000–589.2001.

[fsn3322-bib-0010] Che Man, Y. B. , Z. A. Syahariza , and A. Rohman . 2011 Discriminant analysis of selected edible fats and oils and those in biscuit formulation using FTIR spectroscopy. Food Anal. Methods 4:404–409.

[fsn3322-bib-0011] Commission Regulation . 2002 (EC) No 1774/2002 of the European Parliament and of the Council of 3 October 2002 laying down health rules concerning animal by‐products not intended for human consumption. Official J. Eur. Commun. L 273:1–95.

[fsn3322-bib-0012] De Taeye, B. M. , C. Morisseau , J. Coyle , J. W. Covington , A. Luria , J. Yang , et al. 2010 Expression and regulation of soluble epoxide hydrolase in adipose tissue. Obesity 18:489–498. 19644452 10.1038/oby.2009.227 PMC2864128

[fsn3322-bib-0013] ECSSC (European Conference on Solid State Chemistry) . 1999 Opinion on the safety of tallow derivatives from cattle tallow. Available at http://ec.europa.eu/food/fs/sc/ssc/out359_en.pdf (accessed 14 July 2014).

[fsn3322-bib-0014] Gondret, F. , B. Guével , E. Com , A. Vincent , and B. Lebret . 2012 A comparison of subcutaneous adipose tissue proteomes in juvenile piglets with a contrasted adiposity underscored similarities with human obesity. J. Proteomics 75:949–961. 22061664 10.1016/j.jprot.2011.10.012

[fsn3322-bib-0015] Hsieh, Y.H.P . 2006 Meat species identification Pp. 30/1–30/19 *in* Hui Y. H., ed. Handbook of Food Science Technology, and Engineering. CRC Press, Boca Raton, FL.

[fsn3322-bib-0016] Kershaw, E. E. , and J. S. Flier . 2004 Adipose tissue as an endocrine organ. J. Clini. Endocrinol. Metabol. 89:2548–2556. 10.1210/jc.2004-0395 15181022

[fsn3322-bib-0017] Marikkar, J. M. N. , H. M. Ghazali , Y. B. Che Man , and O. M. Lai . 2002 The use of cooling and heating thermograms for monitoring of tallow, lard and chicken fat adulterations in canola oil. Food Res. Int. 35:1007–1014.

[fsn3322-bib-0018] Marikkar, J. M. N. , H. M. Ghazali , Y. B. Che Man , T. S. G. Peiris , and O. M. Lai . 2005 Distinguishing lard from other animal fats in admixtures of some vegetable oils using liquid chromatographic data coupled with multivariate data analysis. Food Chem. 91:5–14.

[fsn3322-bib-0019] Martín, I. , T. García , V. Fajardo , I. López‐Calleja , P. E. Hernández , I. González , et al. 2007 Species‐specific PCR for the identification of ruminant species in feedstuffs. Meat Sci. 75:120–127. 22063419 10.1016/j.meatsci.2006.06.019

[fsn3322-bib-0020] Montiel‐Sosa, J. F. , E. Ruiz‐Pesini , J. Montoya , P. Roncalés , M. J. López‐Pérez , and A. Pérez‐Martos . 2000 Direct and highly species‐specific detection of pork meat and fat in meat products by PCR amplification of mitochondrial DNA. J. Agric. Food Chem. 48:2829–2832. 10898631 10.1021/jf9907438

[fsn3322-bib-0021] Motoyama, M. , M. Ando , K. Sasaki , and H. O. Hamaguchi . 2010 Differentiation of animal fats from different origins: use of polymorphic features detected by Raman spectroscopy. Appl. Spectrosc. 64:1244–1250. 21073793 10.1366/000370210793335070

[fsn3322-bib-0022] Rosenow, A. , T. N. Arrey , F. G. Bouwman , J. P. Noben , M. Wabitsch , E. C. M. Mariman , et al. 2010 Identification of novel human adipocyte secreted proteins by using SGBS cells. J. Proteome Res. 9:5389–5401. 20681635 10.1021/pr100621g

[fsn3322-bib-0023] Sajic, T. , G. Hopfgartner , I. Szanto , and E. Varesio . 2011 Comparison of three detergent‐free protein extraction protocols for white adipose tissue. Anal. Biochem. 415:215–217. 21565151 10.1016/j.ab.2011.04.023

[fsn3322-bib-0024] Szabó, A. , H. Fébel , L. Sugár , and R. Romvári . 2007 Fatty acid regiodistribution analysis of divergent animal triacylglycerol samples ‐ a possible approach for species differentiation. J. Food Lipids 14:62–77.

[fsn3322-bib-0025] Vaclavik, L. , V. Hrbek , T. Cajka , B. A. Rohlik , P. Pipek , and J. Hajslova . 2011 Authentication of animal fats using direct analysis in real time (DART) ionization ‐ mass spectrometry and chemometric tools. J. Agric. Food Chem. 59:5919–5926. 21526761 10.1021/jf200734x

[fsn3322-bib-0026] Zasadny, R. , and K. Kwiatek . 2006 Validation study of a new procedure for measuring insoluble impurities in animal fat. J. Anim. Feed Sci. 15:337–344.

